# Vascular Disease, Non‐Steroidal Anti‐Inflammatory Drugs and Risk of Parkinson's Disease

**DOI:** 10.1002/acn3.70489

**Published:** 2026-07-24

**Authors:** Clare Wotton, Carol Hermon, Sarah Floud, Gillian Reeves, Isobel Barnes

**Affiliations:** ^1^ Cancer Epidemiology Unit, Nuffield Department of Population Health University of Oxford England UK

**Keywords:** cerebrovascular disease, Ischaemic heart disease, non‐steroidal anti‐inflammatory drugs, Parkinson's disease, vascular disease

## Abstract

**Objectives:**

A history of vascular disease has been shown to be associated with an increased risk of Parkinson's disease, but this may be due to reverse causation. In addition, non‐steroidal anti‐inflammatory drugs are thought to reduce Parkinson's disease risk, but evidence is inconsistent and confounding by prior vascular disease is possible. Our objective was to examine these associations in the UK prospective Million Women Study.

**Methods:**

A total of 819,575 women were followed from median year 2001 for first hospital admission or death from Parkinson's disease. A history of ischaemic heart disease and of cerebrovascular disease was ascertained through hospital admission records and self‐report at baseline. Use of the two non‐steroidal anti‐inflammatory drugs, aspirin and ibuprofen, was ascertained through self‐report at baseline. Cox regression was used to estimate adjusted hazard ratios.

**Results:**

During an average follow‐up of 18 years, 10,364 cases of Parkinson's disease occurred. Women with prior ischaemic heart disease had an increased risk of Parkinson's disease (Hazard ratio 1.45, 95% confidence interval 1.35–1.54), as did women with prior cerebrovascular disease (1.51, 1.32–1.73); both associations were still pronounced 10 or more years after baseline. There was little association of aspirin use with risk of Parkinson's disease (1.06, 1.00–1.13) after adjustment for vascular disease, and no association with ibuprofen use (1.04, 0.97–1.11).

**Interpretation:**

Ischaemic heart disease and cerebrovascular disease are both positively associated with subsequent risk of Parkinson's disease and these associations are unlikely to be solely due to reverse causation. In contrast, use of non‐steroidal anti‐inflammatory drugs is not materially associated with risk.

## 1 Introduction

Parkinson's disease (PD) is a progressive neurodegenerative condition characterised by motor symptoms, including bradykinesia, rigidity, rest tremor, and postural instability [1, 2]. Diagnosis typically occurs with the onset of motor symptoms, but this can be preceded by a prodromal phase lasting many years. During this phase, individuals may experience a range of non‐motor symptoms such as sleep disorders, depression, and constipation [3].

Genetic factors contribute to PD in only about 10% of cases, suggesting that other factors are likely involved in its development [2]. The main finding to date has been a decreased risk of PD associated with cigarette smoking [4, 5]. Several cohort studies have also found that prior vascular disease, particularly stroke, is associated with an increased risk of PD [6, 7, 8, 9]. Given the long prodromal phase of PD and that vascular disease may sometimes be an early manifestation of PD occurring before the onset of motor symptoms [10], it is difficult to disentangle cause from effect. As a result, the observed associations of prior vascular disease with PD risk may be biassed by reverse causation, especially in the short term. However, the longest average follow‐up among previous studies was 11.5 years, which is not long enough to examine this potential bias.

It has been hypothesised that the use of non‐steroidal anti‐inflammatory drugs (NSAIDs) may reduce the risk of PD by reducing inflammation in the brain, but the evidence to date is inconsistent. A meta‐analysis of 8 observational studies found aspirin use was associated with an increase in the risk of PD, while ibuprofen use was associated with a decrease in risk [11]. In contrast, subsequent individual studies have found no association with aspirin use and little association with ibuprofen use [12, 13, 14, 15]. This discrepancy may be because the later studies, unlike most of those included in the meta‐analysis, accounted for reverse causation whereby early symptoms of PD alter NSAID use. It may also be partly due to later studies adjusting for prior vascular disease, which is potentially a major confounder of any association between aspirin and PD as individuals with prior vascular disease are often prescribed aspirin for secondary prevention [16].

We aimed to assess evidence for a causal association between prior vascular disease and use of the two NSAIDs, aspirin and ibuprofen, and the risk of PD in 819,575 women with a mean of 18 years of follow‐up, taking careful account of reverse causation and potential confounding.

## 2 Methods

### 2.1 Study

We analysed data from the Million Women Study, a population‐based prospective cohort study of UK women [17, 18]. Around 1.3 million women were recruited between 1996 and 2001 through National Health Service (NHS) breast cancer screening centres in England and Scotland. Participants gave written consent for follow‐up through medical records. The study was given ethical approval by the East of England‐Cambridge South Research Ethics Committee. Information on data access is provided on the Million Women Study website [19].

Women completed a questionnaire at recruitment and reported personal and health information, and subsequently were asked to complete re‐survey questionnaires at ≈3–5 years intervals. A re‐survey questionnaire completed about 3 years after recruitment in median year 2001 included a question on NSAID use for the first time and was the baseline for the analyses presented here. Specifically, participants were asked ‘Have you taken any medications for most of the last 4 weeks?’ with a list of 15 medications including the two NSAIDs aspirin and ibuprofen. We refer to reported use for most of the last 4 weeks as regular use and categorised women as regular or non‐regular users of each NSAID.

Women's questionnaire data were linked electronically to medical records, including hospital admission records and death registrations. Hospital admission records included up to 20 diagnoses per admission in England and up to 6 per admission in Scotland, all coded according to the World Health Organisation's International Classification of Diseases (ICD). The underlying cause of mortality on death registrations was also coded using the ICD.

We defined women as having prior cerebrovascular disease if, at baseline, they reported having been diagnosed with stroke or transient ischaemic attack or had a previous hospital admission for stroke (ICD‐10 I60‐I69, ICD‐9 430–438). Likewise, we defined women as having prior ischaemic heart disease if, at baseline, they reported having been diagnosed with, or treated for, heart disease or had a previous hospital admission for ischaemic heart disease (ICD‐10 I20‐I25, ICD‐9 410–414). Henceforward, cerebrovascular disease and ischaemic heart disease will be collectively referred to as vascular disease.

Follow‐up for these analyses was provided via electronic linkage to hospital admission records and death registrations. Our primary outcome was the first hospital admission with any recorded diagnosis of PD (ICD‐10 G20) or death with PD recorded as the underlying cause.

### 2.2 Statistical Analysis

Our analyses excluded women who reported having had a diagnosis of PD on the recruitment questionnaire, or who had a hospital admission record with any mention of a diagnosis of PD (ICD‐10 G20, ICD‐9 332.0) or secondary parkinsonism (ICD‐10 G21, ICD‐9 332.1) prior to baseline. We also excluded women if they did not answer the above question regarding medication use, or could not be followed‐up as they had emigrated prior to baseline.

We used Cox proportional hazard models to estimate hazard ratios (HRs) and 95% confidence intervals (CIs) for PD according to prior vascular disease and, separately, according to NSAID use. Attained age was used as the underlying time variable. Women contributed person‐years from baseline up to the date of first admission with a diagnosis of PD or secondary parkinsonism, date of death, date of emigration, or 31st December 2021, whichever occurred earliest.

We stratified our analyses by geographical region of recruitment (10 regions corresponding to the regional health authorities) and adjusted for age at baseline (< 55, 55–57, 58–63, and 63+ years), area deprivation quintile at recruitment (based on the Townsend index [20]), educational qualifications (secondary/tertiary, technical/none), smoking at baseline (never, past, current), alcohol consumption at baseline (0, 1–6, 7+ drinks a week), body mass index at baseline (< 20, 20–24.9, 25–29.9, 30+ kg/m^2^), physical activity at baseline (once a week or less, more than once a week), and a history of diabetes at baseline. As data for adjustment variables was relatively complete (< 7.5% missing for all variables except alcohol consumption [12.5%]), missing values were allocated to a separate category.

We treated prior vascular disease and NSAID use as fixed baseline exposures rather than time‐dependent exposures because a main aim of this study was to examine whether any observed associations with PD risk could be explained by reverse causation. Updating a woman's exposure status with respect to prior vascular disease on the basis of post‐baseline events could exacerbate reverse causation bias if, as has been suggested, such events are sometimes early manifestations of PD. Similarly, updating NSAID use during follow‐up could exacerbate reverse causation bias if post‐baseline changes in NSAID use were in response to early symptoms of PD.

To assess the potential impact of reverse causation, we repeated our analyses according to follow‐up period (< 10, 10+ years from baseline). Associations for the period 10 or more years after baseline are expected to be less susceptible to reverse causation because the baseline exposure is more likely to have preceded the prodromal phase of PD.

We conducted several additional analyses to further examine the evidence for causal associations. First, to assess the potential for residual confounding by smoking, which is known to be strongly associated with an increased risk of vascular disease and a decreased risk of PD, we repeated our analyses of prior vascular disease among women who reported having never smoked. Second, to assess the potential for confounding by prior vascular disease, we repeated our analyses of NSAID use with additional adjustment for prior vascular disease and among women without prior vascular disease. Finally, we assessed the impact of additional adjustment for morbidities which may be related to vascular disease or NSAID use, specifically high blood pressure, high cholesterol, and rheumatoid arthritis and osteoarthritis.

## 3 Results

After exclusions (1119 women had PD or secondary parkinsonism diagnosed before baseline, 45,741 women had missing information on medication use and 100 women emigrated prior to baseline), our analyses included 819,575 women. Over a mean of 18.2 (SD 4.3) years of follow‐up, there was a first mention of PD in hospital admission data or on a death certificate in 10,364 (1.3%) women: 10,234 as a hospital record and 130 on the death certificate.

Table 1 shows the characteristics of the women included in these analyses according to their history of vascular disease and according to their reported use of NSAIDs. There was substantial variation in women's characteristics by history of vascular disease. Women with either prior ischaemic heart disease or prior cerebrovascular disease were more likely to be older, more deprived, less educated, a past/current smoker, drink less alcohol, have a higher BMI, exercise less, and to have had diabetes.

**TABLE 1:** Personal characteristics for women included in the analysis by prior vascular disease and regular use of non‐steroidal anti‐inflammatory drugs.

Personal characteristic (% unless otherwise stated)	Ischaemic heart disease		Cerebrovascular disease		Aspirin		Ibuprofen	
	Yes	No	Yes	No	Yes	No	Yes	No
Number of women	51,857	767,718	11,307	808,268	88,436	731,139	76,228	743,347
Sociodemographic and lifestyle factors								
Age in years, mean (SD)	61.9 (5.2)	59.4 (4.9)	61.9 (5.3)	59.5 (4.9)	61.6 (5.2)	59.3 (4.9)	59.2 (4.9)	59.6 (5.0)
Most deprived quintile	26	16	26	17	21	16	18	17
Technical or no educational qualifications	65	54	64	55	60	54	58	55
Ever smoker	56	46	53	46	51	46	51	46
Number of alcohol drinks per week, mean (SD)	3.2 (5.0)	4.5 (5.8)	3.1 (5.1)	4.4 (5.8)	3.9 (5.7)	4.4 (5.8)	4.5 (5.9)	4.4 (5.7)
BMI in kg/m2, mean (SD)	27.7 (5.4)	26.1 (4.5)	27.3 (5.4)	26.1 (4.6)	27.2 (5.1)	26.0 (4.5)	26.9 (4.9)	26.1 (4.6)
Strenuous exercise at least once a week	32	42	30	42	37	42	40	42
Prior disease								
Ischaemic heart disease	—	—	—	—	32	3	5	6
Cerebrovascular disease	—	—	—	—	8	1	1	1
Diabetes	11	3	11	4	11	3	4	4

Abbreviation: SD, standard deviation.

There was also substantial variation in the characteristics of women by regular use of NSAIDs. Women who used aspirin were more likely to be older, more deprived, less educated, a past/current smoker, consume less alcohol, have a higher BMI, exercise less, have had diabetes and, in particular, have had vascular disease. Women who reported using ibuprofen were more likely to be a past/current smoker and to have a higher BMI.

**FIGURE 1:**
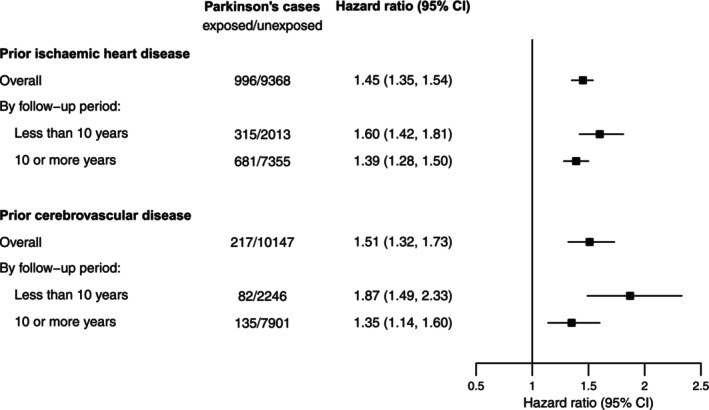
Hazard ratios (95% CIs) for Parkinson's disease in relation to prior vascular disease. Hazard ratios stratified by region and adjusted for age at baseline, deprivation quintile, education, smoking, alcohol consumption, body mass index, physical activity and depression. CI, confidence interval.

Figure 1 presents the HRs for PD in relation to prior vascular disease. Women with prior ischaemic heart disease had an increased risk of PD (HR 1.45, 95% CI 1.34–1.54). When this association was examined separately according to follow‐up period, it was greater during the first 10 years after baseline (1.60, 1.42–1.81) but remained pronounced 10 or more years after baseline (1.39, 1.28–1.50). Likewise, women with prior cerebrovascular disease had an increased risk of PD (1.51, 1.32–1.73), and this association was greater in the first 10 years after baseline (1.87, 1.49–2.33), but was still marked in the period 10 or more years after baseline (1.35, 1.14–1.60). Similar associations were seen among women who reported having never smoked (Table S1), suggesting little residual confounding.

**FIGURE 2:**
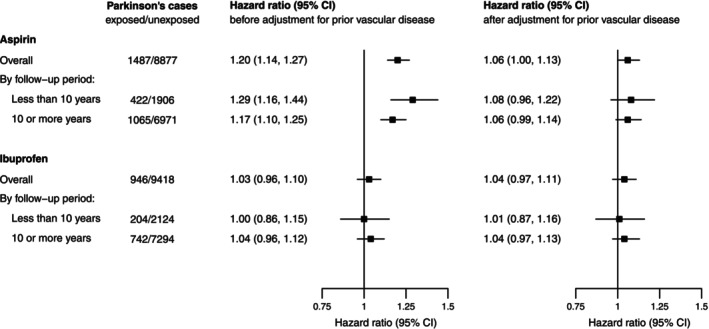
Hazard ratios (95% CIs) for Parkinson's disease in relation to regular use of nonsteroidal anti‐inflammatory drugs. Hazard ratios stratified by region and adjusted for age at baseline, deprivation quintile, education, smoking, alcohol consumption, body mass index, physical activity and depression. CI, confidence interval.

Figure 2 presents the HRs for PD in relation to regular use of NSAIDs. Women who were regular users of aspirin were at an increased risk of PD compared to women who were non‐regular users (1.20, 1.14–1.27). However, after additional adjustment for prior vascular disease this association largely disappeared (1.06, 1.00–1.13). There was no association with regular use of ibuprofen either before or after adjustment for prior vascular disease. A similar lack of association was observed in the follow‐up periods less than 10 years and 10 or more years after baseline. Furthermore, the associations observed after additional adjustment for prior vascular disease were similar to those among women without a history of vascular disease (Table S2), suggesting little residual confounding.

The results of the analysis of the effects of additional adjustment for morbidities related to prior vascular and/or NSAID use are given in Table S3. Additional adjustment for high blood pressure, high cholesterol, and rheumatoid and osteoarthritis made no substantive difference to the estimated hazard ratios for PD related to prior vascular disease or NSAID use.

## 4 Discussion

In this prospective UK cohort study of 819,575 women, among whom 10,364 had a first record of PD during a mean 18 years of follow‐up, we found that both prior ischaemic heart disease and prior cerebrovascular disease were associated with a marked increase in the risk of PD. We also found no association of ibuprofen use with the risk of PD and, after accounting for the strong confounding effect of prior vascular disease, little association of regular aspirin use with the risk of PD.

The 56% increase in the risk of PD observed here in women with prior ischaemic heart disease is larger than the associations observed in previous studies, including two retrospective cohort studies based on health insurance records from the US [6] and South Korea [7], and a prospective cohort study conducted in China [8]. The US study, which included 5774 cases of PD, reported prior ischaemic heart disease was associated with a 28% increase in the risk of PD (HR 1.28, 95% CI 1.22–1.33). The Chinese study, which included 696 cases of PD, reported prior ischaemic heart disease was similarly associated with a 20% increase in risk (1.20, 0.89–1.61); however, perhaps due to the relatively small number of cases, this association was not statistically significant. The South Korean study included 9924 cases and reported a small increase in the risk of PD (1.09, 1.02–1.17) associated with prior ischaemic heart disease.

We found that women with prior cerebrovascular disease had a 49% increase in the risk of PD, which is consistent with the strong positive associations observed in previous studies. The US retrospective cohort study [6] reported prior stroke was associated with a 55% increase in the risk of PD (1.55, 1.39–1.72), and the Chinese prospective cohort study [8] reported prior stroke was associated with an almost two‐fold increase in the risk of PD (1.94, 1.39–2.69). In addition, a prospective longitudinal analysis of South Korean health insurance records [9], which included 5574 cases of PD, reported individuals with a history of stroke had a 67% increase in the risk of PD (1.67, 1.57–1.78).

It has previously been suggested that the apparent association of vascular disease with subsequent PD is due to reverse causation whereby vascular disease may be an early manifestation of PD which occurs before the onset of the motor symptoms typically used to diagnose the condition [10]. However, our findings are unlikely to be solely due to reverse causation as we observed that the associations with vascular disease remained marked for follow‐up of 10 or more years. Additionally, it is unlikely that our findings are due to residual confounding by smoking, as we also observed marked associations with vascular disease among women who reported never having smoked.

The causal mechanism by which vascular disease may lead to PD is unclear. One hypothesis is that cerebrovascular disease leads to an environment in the brain that facilitates the development of PD [9]. However, our findings that both cerebrovascular and ischaemic heart disease are associated with an increased risk of PD suggest a more systemic mechanism such as a shared pathogenesis. Mitochondrial dysfunction, oxidative stress and inflammation are thought to contribute to the development and progression of atherosclerosis, which increases the risk of both ischaemic heart disease and cerebrovascular disease [21]. These same three processes are also thought to play a key role in the aggregation of alpha‐synuclein that, in turn, can form the Lewy bodies characteristic of PD [22].

The associations of the two NSAIDs, ibuprofen and aspirin, with the risk of PD were summarised by a meta‐analysis published in 2011 which minimised recall bias by excluding studies with information on NSAID use collected after PD diagnosis [11]. The meta‐analysis found ibuprofen use was associated with a 27% decrease in PD risk (0.73, 0.63–0.85) based on 5 studies with a total of 2168 cases. This contrasts with our finding of no association with ibuprofen use based on about five times as many cases. However, since the meta‐analysis, 3 relatively large studies have been published all with findings similar to ours. These include a Norwegian retrospective cohort study [12], and two case–control studies based on health and pharmaceutical records from Denmark [13] and the UK [14]. The Norwegian study, which included 7580 cases, and the Danish study, which included 1931 cases, both reported no association with ibuprofen use. The UK study included 4026 cases and reported ibuprofen was associated with a small increase in risk (1.09, 1.01–1.18).

We found regular aspirin use was associated with a 6% increase in the risk of PD. This is broadly consistent with the meta‐analysis [11] which reported a 12% increase (1.12, 1.01–1.23) based on 8 studies with a total of 2719 cases. However, only 3 studies in the meta‐analysis adjusted for prior vascular disease, which we found largely attenuated the association between aspirin use and the risk of PD. Since the meta‐analysis, 3 studies have been published including the Danish [13] and UK case–control studies [14], and a US nested case–control study [15], which included 616 cases. All three studies reported no association between aspirin use and the risk of PD after adjusting for vascular disease and accounting for reverse causation.

The strengths of this study include its prospective design, the large sample size (10,364 incident cases of PD), long follow‐up (a mean of 18 years), completeness of follow‐up, and the availability of comprehensive information on major confounders. Due to the large number of cases and the length of follow‐up, we were able to account for the possibility of reverse causation by examining the long‐term risk of PD. We were also able to carefully adjust for major potential confounding of the association between aspirin and risk of PD (by prior vascular disease), and of the association between vascular disease and PD risk (by smoking).

A limitation of this study is that we used hospital admission and death registration data to ascertain PD. Women with hospital admission data may represent a subset of cases with more severe disease. However, since an estimated 87% of women with a primary care record of PD go on to have a hospital record of PD within the next 10 years, the vast majority of cases will be picked up by hospital admission data. Additionally, we found that this estimate varied little by vascular disease or NSAID use, suggesting that differential ascertainment is unlikely to have biassed our findings.

Another limitation is the potential for some degree of misclassification in the measurement of prior vascular disease, based on hospital admission records and self‐report, and of regular NSAID use, which was ascertained from self‐reported medication use. However, findings from validation studies within the Million Women Study suggest that substantial misclassification is unlikely. Diagnoses of vascular disease recorded in hospital admission data have been shown to agree closely with those recorded in primary care records [23], and self‐reported treatment for cardiovascular disease has been shown to have good agreement with prescription records [24]. Although self‐reported NSAID use has not been directly validated, self‐reported use of other medications, such as menopausal hormone therapy, has also been shown to have good agreement with prescription records [24]. Furthermore, any non‐differential exposure misclassification would be expected to bias associations towards the null.

The categorisation of regular NSAID use according to whether women reported use during most of the 4 weeks prior to baseline is also a limitation, as we were unable to assess associations with duration, dose, or frequency for the majority of women, although the accuracy of recall over such a short period is likely to be relatively good. More detailed information about aspirin use was collected 9 years after baseline and the average duration of aspirin use among regular users was 5 years, suggesting longer‐ rather than shorter‐term use of NSAIDs by women in this population.

Our findings provide large‐scale prospective evidence for a pronounced increase in risk of PD in women with ischaemic heart disease and in women with cerebrovascular disease. We also provide evidence that there is little or no association of ibuprofen or aspirin use with the risk of PD.

## Author Contributions

S.F. and G.R. are the co‐principal investigators of the Million Women Study, and both were involved in the design of the study. I.B., C.H., and C.W. analysed the data, and I.B. prepared the tables and figures. C.W. drafted the original manuscript, and I.B., S.F., C.H., and G.R. contributed towards subsequent versions.

## Funding

This Million Women Study has received funding from the Medical Research Council (UKRI1464) and Cancer Research UK (C16077/A29186).

## Conflicts of Interest

The authors declare no conflicts of interest.

## Supporting information


**Table S1:** Hazard ratio (95% CIs) for Parkinson's disease in relation to prior vascular disease among women who reported having never smoked.
**Table S2:** Hazard ratio (95% CIs) for Parkinson's disease in relation to regular use of nonsteroidal anti‐inflammatory drugs among women without prior vascular disease.
**Table S3:** Hazard ratios (95% CIs) for Parkinson's disease in relation to prior vascular disease and in relation to regular use of nonsteroidal anti‐inflammatory drugs among women after additional adjustment for high blood pressure, high cholesterol, rheumatoid arthritis and osteoarthritis.

## Data Availability

Data from the Million Women Study are available to bona fide researchers in accordance with the Million Women Study Data Access Policy (https://www.ceu.ox.ac.uk/research/the‐million‐women‐study/data‐access‐and‐sharing/data‐access‐policy). Further information is available from the corresponding author upon request.
