# Supplementary material for: Vascular Disease, Non‐Steroidal Anti‐Inflammatory Drugs and Risk of Parkinson's Disease
Source: Ann Clin Transl Neurol. 2026 Jul 24:10.1002/acn3.70489. Online ahead of print. doi: 10.1002/acn3.70489 (PMC13398127; doi:10.1002/acn3.70489)
Supplement: Supplementary file 1 — Table S1: Hazard ratio (95% CIs) for Parkinson's disease in relation to prior vascular disease among women who reported having never smoked. Table S2: Hazard ratio (95% CIs) for Parkinson's disease in relation to regular use of nonsteroidal anti‐inflammatory drugs among women without prior vascular disease. Table S3: Hazard ratios (95% CIs) for Parkinson's disease in relation to prior vascular disease and in relation to regular use of nonsteroidal anti‐inflammatory drugs among women after additional adjustment for high blood pressure, high cholesterol, rheumatoid arthritis and osteoarthritis. [file ACN3-9999-0-s001.pdf]

Supplementary Table 1. Hazard ratio (95% CIs) for Parkinson's disease in relation to prior vascular disease among women who reported having never smoked.

Exposure	Parkinson's disease cases exposed/unexposed	Hazard ratio (95% CI)
**Prior ischaemic heart disease**		
Overall	545/5661	1.44 (1.32, 1.58)
By follow-up period:		
<10 years	163/1235	1.49 (1.26, 1.77)
10+ years	382/4426	1.42 (1.28, 1.58)
**Prior cerebrovascular disease**		
Overall	108/6098	1.40 (1.16, 1.69)
By follow-up period:		
<10 years	31/6098	1.28 (0.90, 1.84)
10+ years	135/7901	1.43 (1.14, 1.80)

Hazard ratios stratified by region and adjusted for age at baseline, deprivation quintile, education, smoking, alcohol consumption, body mass index, physical activity and diabetes. CI, confidence interval.

Supplementary Table 2. Hazard ratio (95% CIs) for Parkinson's disease in relation to regular use of nonsteroidal anti-inflammatory drugs among women without prior vascular disease.

**Exposure**	**Parkinson's disease cases exposed/unexposed**	**Hazard ratio (95% CI)**
**Regular use of aspirin**		
Overall	799/8417	1.07 (1.00, 1.15)
By follow-up:		
<10 years	189/1767	1.03 (0.89, 1.20)
10+ years	610/6650	1.09 (1.00, 1.18)
**Regular use of ibuprofen**		
Overall	862/8354	1.04 (0.97, 1.12)
By follow-up:		
<10 years	173/1783	0.99 (0.84, 1.15)
10+ years	689/6571	1.06 (0.98, 1.14)

Hazard ratios stratified by region and adjusted for age at baseline, deprivation quintile, education, smoking, alcohol consumption, body mass index, physical activity and diabetes. CI, confidence interval.

Supplementary Table 3. Hazard ratios (95% CIs) for Parkinson's disease in relation to prior vascular disease and in relation to regular use of nonsteroidal anti-inflammatory drugs among women after additional adjustment for high blood pressure, high cholesterol, rheumatoid arthritis and osteoarthritis.

Exposure	Parkinson's disease cases exposed/unexposed	Relative risk (95% CI)
**Prior ischaemic heart disease**		
Overall	996/9368	1.42 (1.33, 1.52)
By follow-up period:		
<10 years	315/2013	1.56 (1.38, 1.77)
10+ years	681/7355	1.37 (1.26, 1.49)
**Prior cerebrovascular disease**		
Overall	217/10147	1.44 (1.25, 1.65)
By follow-up period:		
<10 years	82/2246	1.74 (1.39, 2.18)
10+ years	135/7901	1.30 (1.09, 1.54)
**Regular aspirin use**		
Overall	1487/8877	1.07 (1.01, 1.14)
By follow-up period:		
<10 years	422/1906	1.09 (0.97, 1.23)
10+ years	1065/6971	1.07 (0.99, 1.15)
**Regular ibuprofen use**		
Overall	946/9418	1.01 (0.95, 1.08)
By follow-up period:		
<10 years	204/2124	0.97 (0.84, 1.12)
10+ years	742/7294	1.02 (0.95, 1.11)

Hazard ratios stratified by region and adjusted for age at baseline, deprivation quintile, education, smoking, alcohol consumption, body mass index, physical activity, diabetes, high blood pressure, high cholesterol, rheumatoid arthritis osteoarthritis, and, in the case aspirin and ibuprofen use, prior vascular disease. CI, confidence interval
